# Opsonins and Vaccines as Applied to Surgical Therapy

**Published:** 1907-09

**Authors:** D. H. Bergey

**Affiliations:** Assistant Professor of Bacteriology, University of Pennsylvania


### Opsonins and Vaccines as Applied to Surgical Therapy.

By D. H. BERGEY, M.D.
Assistant Professor of Bacteriology, University of Pennsylvania.

[ *University of Pennsylvania Medical Bulletin.* ]

THE phagocytic power of the leukocytes was discovered by Leber (1888), but its great importance in overcoming infection was made apparent through the researches of Metchnikoff (1892). On the basis of the phagocytic power of the leukocytes Metchnikoff formulated his theory of immunity which has ever since stood in opposition to the more generally accepted theories, promulgated in Germany and elsewhere, in which the influence of the phagocytes is overshadowed by the destructive powers of the fluid portion of blood serum.

It was not until the discovery of the opsonins by Wright and Douglas (1902) and their demonstration of the important relation of these substances in the blood to the phagocytic power of the leukocytes that the full significance of this leukocytic function was comprehended by scientific investigators.

Though Metchnikoff and those adhering to the French school had for a long time proclaimed what appeared to be extravagant claims for the activity of the leukocytes in overcoming disease, notwithstanding the very generally accepted functions of forces evidently originating and existing outside the leukocytes, the discovery of the opsonins by Wright and Douglas attracted renewed attention to the teaching of Metchnikoff. While the French school regarded the increased phagocytic powers of the leukocytes in immune animals over those exhibited in normal animals, as being due to a direct stimulation of the phagocytic powers of the leukocyte through an immune substance, Wright and Douglas demonstrated that in reality the immune substances—the opsonins—acted upon the bacteria and prepared them for phagocytosis. The studies of Neufeld and Rimpau (1904) confirmed the opinion of Wright and Douglas that the immune substances—the bacteriotropins, as they called them—exerted their action upon the bacteria and not upon the leukocytes.

Our studies of both natural and acquired immunity have revealed to us several varieties of antibodies or immune substances in the blood in different diseases. The principal antibodies through which disease is prevented or overcome after it has become established are the antitoxins, the agglutinins, the precipitins, the bactericidal amboceptors, and the opsonins.

The antitoxins are the most important weapons of defence which the body employs against bacterial infection in those diseases in which the causative agents have the property of secreting soluble toxins, viz., diphtheria and tetanus. The organisms causing these diseases secrete a soluble toxin by means of which they induce the constitutional symptoms and organic lesions which characterise the diseases which they produce. Diphtheria and tetanus are the only diseases of human beings against which we possess efficient antitoxic sera as protective and therapeutic agents.

In another group of diseases, such as typhoid fever, dysentery, and Asiatic cholera, we are dealing with bacteria which do not generate soluble toxins in large amounts, but which have their poisons in intimate association with their body proteids. In immunity from these diseases the most prominent antibody is the bactericidal amboceptor, which has the power of uniting with the bacteria with one of its haptophore groups, while the other haptophore group unites with the complement of the blood and thus makes the destruction of the bacteria possible. These diseases are also characterised by the high agglutinating power of the blood serum upon the invading bacteria, a function which no doubt is of great importance in overcoming the infection.

In still another group of diseases we find that the blood serum acquires neither a high antitoxic, bactericidal, or agglutinating power, but that in these infections the opsonins are the most important antibodies that have thus far been discovered. The diseases which appear to belong to this class are those due to the streptococci, staphylococci, pneumococci, and bacterium tuberculosis in particular, while Malta fever, plague, gonorrhea, cerebrospinal fever, and anthrax are all of a similar character in respect to the immunity which results through recovery from the particular disease. The bacteria causing these diseases also produce poisons which are in intimate association with their own protoplasm, that is, endotoxins.

Opsonins capable of acting upon a great variety of bacteria are found in normal blood of human beings and the domestic animals. Whether these normal opsonins are different for each organism, or whether they possess a character which permits the opsonin to act upon any type of organism, cannot be stated at present. We know that the agglutinins found in normal blood are principally of the nature of common agglutinins, while those resulting from recovery from infection are, in a large part, specific for the particular organism causing the infection. It is probable that in some respects the opsonins, normal and immune respectively, bear a similar relation to the bacteria.

The opsonic power of the blood is increased naturally as the result of recovery from infection, and it can also be increased by systematic immunisation by means of living attenuated bacteria, by dead bacteria, or by means of certain proteid constituents of the bacterial protoplasm. Since Wright has published his important discoveries the dead bacteria are now commonly employed for this purpose.

It has become customary to speak of the suspensions of the attenuated or dead bacteria as "bacterial vaccines," since this term was employed long since by Pasteur to designate his attentuated cultures employed in the protective inoculations against different bacterial diseases. Although the word vaccine is derived from "vacca," a cow, and was first used to designate the material employed to protect against smallpox, the long-established custom of calling the bacterial suspension vaccines has given the term some degree of fixity, besides which it is not altogether inappropriate to call all of the substances used for the purpose of active immunisation or protection against disease by the same name.

The results of the inoculation of the bacterial vaccines are determined by noting their effect upon the opsonic index of the blood serum of the vaccinated individual. By the opsonic index is meant the relative influence of a patient's blood upon the phagocytosis as compared with that of normal individuals.

The opsonic index of an individual's blood serum is determined by mixing in a capillary tube equal parts of the individual's serum, a suspension of leukocytes, and an emulsion of the bacteria against which the index is to be ascertained. At the same time several control tests are made with the same leukocytes and bacterial emulsion and the blood serum of several normal individuals. These several mixtures of leukocytes, bacteria, and serum are then incubated at the body temperature for fifteen minutes and then thin smears are prepared on clean microscopic slides. These smears are dried and stained, when the number of bacteria taken up by 75 to 100 leukocytes in each preparation is enumerated. If, for instance, the average number of bacteria taken up by the leukocytes under the influence of the normal serum is 20, and the average number taken up by the leukocytes under the influence of the patient's serum is 10, then, regarding the index of the normal individual as unity, the index of the patient's blood would be 0.5.

A low opsonic index is regarded as indicating an unusually low degree of resistance against infection or the presence of infection. On the other hand, a high opsonic index is regarded as indicating either a high degree of resistance to infection, probably as the result of recovery from infection by the particular organism, or a low grade of infection in which there is insufficient opportunity for auto-immunisation to completely overcome the infection.

Some American investigators have expressed considerable doubt as to the reliability of the opsonic index as an indicator of the progress of infection, because of the variability of the results they have obtained in the study of the index of normal individuals as well as in the study of the effects of inoculations. Other investigators have not encountered such marked variations in their results, and hence they are inclined to agree with Wright and his associates that, while absolutely concordant results are not to be expected, nevertheless, the results of control tests should not vary more than about 10 per cent. It is well known that some of the investigators who have reported varying results have deviated from some of the details of Wright's technique, and it is probable that in this fact lies the chief cause for the nature of the results they have obtained.

The bacterial vaccines are prepared by cultivating the organisms on the surface of agar-agar and then flushing the bacteria off by means of normal salt solution. These suspensions of the bacteria are subjected to heat—65° to 80° C. for an hour—so as to kill the organisms. Inasmuch as the size of the dose inoculated has an important influence upon the patient and the course of the infection, it becomes necessary to ascertain the number of bacteria in a certain volume of the suspension. This is done by mixing equal quantities of blood and bacteria, and then by counting both erythrocytes and the bacteria in the same number of squares of a blood-counting cell the number of bacteria per cubic centimeter of emulsion can be calculated.

The size of the dose of bacterial emulsion to be inoculated must be governed by the patient's initial opsonic index, the extent of the infection, and also by the general condition of the patient, especially with regard to the presence or absence of fever. In persons with fever the dose should always be smaller than in those without fever. The interspacing of the doses is likewise determined by observing the patient's opsonic index. Immediately following an injection of the vaccine the index falls for a time—the so-called negative phase of Wright—to be followed in a day or two by a rise in the index to, or above, its original level

—the positive phase of Wright. Subsequent inoculations should not be made until the opsonic index has returned to, or above, the initial point.

The extent and duration of the negative phase is influenced to a large degree by the relative size of the dose administered, that is, the larger the dose given the greater is the negative phase, and the more slowly does it give way to the positive phase. Wright directs, however, that in cases where the positive phase is slow in manifesting itself and is not pronounced, it is best not to wait too long before repeating the injection, but under the circumstances he advises the administration of a smaller dose than was given at the first injection.

Wherever it is possible to do so, Wright recommends the use of autogenous cultures in the preparation of the bacterial vaccines in preference to the use of stock cultures. In the treatment of tuberculosis Wright employs Koch's new tuberculin, using 0.004 to 0.06 mgm. of the dry powder at a dose. The number of bacteria to be administered in the treatment of the other bacterial infections ranges from 50,000,000 to 500,000,000, according to circumstances. On general principles it is advisable to begin with the smaller dose and gradually increase the amount injected if the response obtained warrants such a course.

The bacterial vaccines are employed in public health work and in general medicine either as protective agents or as curative agents, and they may be employed by the surgeon on the same broad scope. While the use of the vaccines is of greatest importance to the surgeon in the treatment of certain chronic infections, where operative procedures have proven more or less disappointing, they may also be used to fortify the system against bacterial invasion in, or following, operations where aseptic and antiseptic measures cannot be satisfactorily employed.

The diseases amenable to treatment with the bacterial vaccines are: Furunculosis, carbunculosis, and circumscribed suppurating inflammations due to the staphylococci; chronic inflammations due to streptococci or pneumococci; but especially localised infections due to bacterium tuberculosis, as tuberculous lesions of the skin and bones, tuberculous glands, tuberculous peritonitis, and tuberculosis of the genito-urinary tract. In chronic tuberculous lesions, as in cases of psoas abscess, Gray (1906) directs the evacuation of the caseous material, and then follows this with injections of mixed vaccines consisting of tuberculin and staphylococcus and streptococcus vaccines. In caseous or calcareous lymphatic glands Gray follows the same procedure with gratifying results. In tuberculous lesions where mixed staphylococcus and streptococcus infection does not exist the tuberculin alone suffices to bring about a rapid reduction in the size of the infected glands, or the arrest of the infectious process.

In the treatment of various infectious processes by means of bacterial vaccines the use of certain subsidiary measures is frequently advisable to assist the body in overcoming the infection. Massage may be employed with great benefit during the time the patient is being treated with the vaccines in order to facilitate the more active penetration of the infected area by the blood and lymph carrying the specific opsonin or in reëstablishing the flow of blood and lymph through the part. Measures to induce passive hyperemia of the infected area bring about similar results. Wright has been able to demonstrate that these subsidiary measures may prove positively harmful in the absence of treatment with the vaccines because of the sudden liberation of large quantities of poisonous substances lodging in the infected area.

The bacterial vaccines may also be employed as protective agents in surgical practice where it is evident that satisfactory aseptic and antiseptic measures cannot be instituted, or in cases where the general vitality of the patient is low and it is probable that secondary infection by the pyogenic bacteria will follow.

In operations in the mouth, as for cleft palate, where it is impossible to control secondary infection by the pyogenic organisms, it is advisable to fortify the patient's system by bacterial inoculations so as to increase his opsonic index for staphylococci and streptococci before undertaking the operation.

In diabetics and albuminurics where the general vitality of the body is low it is believed that the success of surgical procedures can be materially enhanced by first raising the individual's opsonic index for the common pyogenic organisms. In a similar way secondary infection of tuberculous abscesses may be avoided, if a course of treatment with bacterial vaccines precedes the opening of the abscess.

In some accident wounds where perfect asepsis is doubtful, and where the mutilation of the tissues makes secondary infection by the pyogenic organisms probable, the use of the vaccines appears to be clearly indicated.

In conclusion, no better advice can be given than is contained in a recent paper by Wright (*Lancet,* December 2, 1905): "It is a matter of great moment, especially in immunisation against tubercle, to employ in every case the smallest doses which will elicit a satisfactory response, to repeat the dose only when the effect of the preceding inoculation is passing off, and to increase the dose only when it becomes clear that the dose previously employed is ceasing to evoke a sufficient immunising response."

